# Cerebral Ketone Body Oxidation Is Facilitated by a High Fat Diet Enriched with Advanced Glycation End Products in Normal and Diabetic Rats

**DOI:** 10.3389/fnins.2016.00509

**Published:** 2016-11-08

**Authors:** Adriano M. de Assis, Jussemara S. da Silva, Anderson Rech, Aline Longoni, Yasmine Nonose, Cendrine Repond, Matheus A. de Bittencourt Pasquali, José C. F. Moreira, Diogo O. Souza, Luc Pellerin

**Affiliations:** ^1^Postgraduate Program in Biological Sciences: Biochemistry, Institute of Basic Health Science (ICBS), Federal University of Rio Grande do SulPorto Alegre, Brazil; ^2^Department of Physiology, University of LausanneLausanne, Switzerland; ^3^Department of Biochemistry, Institute of Tropical Medicine, Federal University of Rio Grande do NorteNatal, Brazil; ^4^Department of Biochemistry, Federal University of Rio Grande do SulPorto Alegre, Brazil

**Keywords:** brain energy metabolism, diabetes mellitus, high fat diet, MCTs, AGEs

## Abstract

Diabetes mellitus (DM) causes important modifications in the availability and use of different energy substrates in various organs and tissues. Similarly, dietary manipulations such as high fat diets also affect systemic energy metabolism. However, how the brain adapts to these situations remains unclear. To investigate these issues, control and alloxan-induced type I diabetic rats were fed either a standard or a high fat diet enriched with advanced glycation end products (AGEs) (HAGE diet). The HAGE diet increased their levels of blood ketone bodies, and this effect was exacerbated by DM induction. To determine the effects of diet and/or DM induction on key cerebral bioenergetic parameters, both ketone bodies (β-hydroxybutyric acid) and lactate oxidation were measured. In parallel, the expression of Monocarboxylate Transporter 1 (MCT1) and 2 (MCT2) isoforms in hippocampal and cortical slices from rats submitted to these diets was assessed. Ketone body oxidation increased while lactate oxidation decreased in hippocampal and cortical slices in both control and diabetic rats fed a HAGE diet. In parallel, the expression of both MCT1 and MCT2 increased only in the cerebral cortex in diabetic rats fed a HAGE diet. These results suggest a shift in the preferential cerebral energy substrate utilization in favor of ketone bodies in animals fed a HAGE diet, an effect that, in DM animals, is accompanied by the enhanced expression of the related transporters.

## Introduction

Alterations in cognitive functions of patients with DM were first described many years ago (Miles and Root, 1922) and have been documented more extensively recently (Frier, 2011; Jacobson et al., 2011; McCrimmon et al., 2012). However, the mechanisms implicated in such mental deterioration are not clear (Ryan et al., 2003; Brands et al., 2006). Interestingly, recent studies have shown that hippocampal metabolism is affected when glycemia is increased (Mcnay and Recknagel, 2011; Duarte et al., 2012). In parallel, a unifying mechanism to explain the complications linked to diabetes has been proposed in which an inhibition of glycolysis by Poly-(ADP-ribose)-polymerase (PARP) generates an increase in glycolytic intermediates (Brownlee, 2005). These intermediates are metabolized through four pathways leading to several diabetic complications and one of these pathways is the formation of dicarbonyl compounds that are precursors of AGEs, offering a putative explanation for cognitive impairments associated with diabetes.

Poor-quality diets are among the lifestyle determinants that affect DM and aging-related diseases and increase the risk of cognitive decline and dementia development in mammals (Mattson, 2012). Elevated dietary fat intake, mainly from saturated fatty acids, contribute to cognitive deficits in rats, implicating both the hippocampus and the cerebral cortex (Kaplan and Greenwood, 1998; Greenwood and Winocur, 2005; Winocur and Greenwood, 2005). In this context, dietary AGEs, a class of oxidative-stressor promoting agents implicated in DM and aging, have been shown to cause cognitive and metabolic disturbances in mice and humans (Cai et al., 2014). In a previous study, (de Assis et al., 2009) showed that non-diabetic rats fed with a high fat diet enriched with AGEs for 12 months had a significant increase in hippocampus DNA damage compared to those fed a control high fat diet.

MCTs belong to a large family of proton-linked carriers that transport monocarboxylic acids such as lactate, pyruvate and ketone bodies (Pierre and Pellerin, 2005). In the central nervous system, various MCTs have been identified and their distribution determined at the cellular level. MCT1 is mostly expressed on astrocytic processes, endothelial cells, and oligodendrocytes in both rodents (Pellerin et al., 1998; Pierre et al., 2000; Baud et al., 2003; Lee et al., 2012) and humans (Froberg et al., 2001; Chiry et al., 2006), whereas MCT2 is a major neuronal transporter (Pierre et al., 2002). It has been shown that 12 weeks of exposure to a high fat diet (without AGEs) that led to doubling of plasmatic β-hydroxybutyrate levels increased both hippocampal and cortical MCT1 and MCT2 levels in mice (Pierre et al., 2007).

Based on these previous observations, the main goal of this work was to evaluate the effects of a high fat diet enriched with AGEs and/or DM induction on bioenergetic parameters and on the MCT (MCT1 and MCT2) expression levels in the hippocampus and cerebral cortex of adult male rats.

## Materials and methods

### Ethics statement

All experiments were approved by the local Ethics Commission (CEUA/UFRGS) under project number 19183 and followed the National Institutes of Health Guide for the Care and Use of Laboratory Animals (NIH publication no. 80-23, revised 1996).

### Chemicals

Alloxan monohydrate, sodium L-lactate, D-glucose and β-actin antibodies were obtained from Sigma-Aldrich (St. Louis, MO, USA). L-[U-^14^C]-Lactate (152 mCi/mmol) were from Amersham International (Little Chalfont, Bucks, UK). [1-^14^C]-3-Hydroxybutyric acid sodium salt (50 mCi/mmol) were obtained from American Radiolabeled Chemicals, Inc. (Saint Louis, MO, USA). Anti-MCT1 and anti-MCT2 antibodies were synthesized and utilized as previously characterized and shown by Pierre et al. (2000).

### Animals, treatments, and diets

Adult male Wistar rats (90 days old) from the Central Animal Facility of the Department of Biochemistry, ICBS, UFRGS were maintained under a standard dark/light cycle (the lights were on between 7:00 a.m. and 7:00 p.m.) at a temperature of 22 ± 2°C.

Alloxan causes specific damages in pancreatic beta cells by favoring the production of hydroxyl radicals (Wilson et al., 1984), while not affecting the glucagon-producing alpha cells (Aleeva et al., 2002). The optimal dosage regimen for intraperitoneally administered alloxan is a single high dose between 150 and 200 mg/kg (Federiuk et al., 2004; de Assis et al., 2015). Although most rats developed type-1 DM after administration of alloxan, a few rats developed type-2 DM, characterized by stable high blood glucose values, with normal ketone concentration (Federiuk et al., 2004).

The rats were divided into 2 groups (*n* = 20 per group) after 8 h of fasting. One group received an intraperitoneal (i.p.) administration of alloxan (150 mg.kg^−1^) diluted in saline (0.9% NaCl) to induce diabetes mellitus, and the other group received saline. After 1 week, glycemia in rats in a fasted state (8 h) was measured. Only animals with a glucose concentration of 15–25 mmol/l were included in the study. After confirming the induction of diabetes (hyperglycemia) by alloxan, each group was subdivided into 2 sub-groups (*n* = 10 per group), as follows: (i) groups that received standard laboratory rat chow and (ii) HAGE–groups that received a high fat diet, which was enriched with AGEs by heating the diet for 60 min at 180°C. The heating regimen of the diets was based on (de Assis et al., 2012), who reported a high AGE content (~1 U/μg) in a heated high fat diet. During the 4-week dietary treatments, the animals had free access to food and water. In this study, we chose to evaluate the effects of a relatively short-term (4 weeks) period of diet plus diabetes induction. It appears to be an initial period during which the effects on metabolism emerge and are not so harmful. This may represent perhaps an optimal time for future therapeutic interventions (de Assis et al., 2012). More details about the diet composition are presented in Table 1.

**Table 1:** **Composition of control and HAGE diets**.

**Composition**	**Control diet (%)**	**HAGE diet (%)**
Commercial bran	–	20.5
Soy Protein Isolate^a^	17.0	15.9
Corn Starch	65.5	–
Sucrose	5.0	20.0
Vitamin mix^b^	1.0	1.0
Mineral salt mix^c^	4.0	2.0
DL-Methionin^d^	0.3	0.3
DL-Lysine^e^	0.3	0.3
Soy Oil	5.0	1.0
Lard	–	39.0

a *Soy Protein Isolate, purity 97% (from Solae, Esteio, Brazil)*.

b *Vitamin mixture: mg/100g of diet (from Roche, São Paulo, Brazil): vitamin A (retinyl acetate), 4; vitamin D (cholecalciferol), 0.5; vitamin E (DL-α-tocopheryl acetate), 10; menadione, 0.5; choline, 200; PABA, 10; inositol,10; niacine (nicotinic acid), 4; pantothenicacid (calcium D- pantothenate), 4; riboflavin, 0.8; thiamin (thiamine hydrochloride), 0.5; pyridoxine (pyridoxine hydrochloride), 0.5; folic acid, 0.2; biotin [D-(+)- biotin], 0.04; vitamin B12, 0.003*.

c *Mineral salt mixture: mg/100 g of diet (from Roche, São Paulo, Brazil): NaCl, 557; KI,3.2; KH_2_PO_4_, 1556; MgSO_4_, 229; CaCO_3_, 1526; FeSO_4_–7H_2_O, 108; MnSO_4_–H_2_O, 16; ZnSO_4_–7H_2_O, 2.2; CuSO_4_–5H_2_O, 1.9; CoCl–6H_2_O, 0.09*.

d *D-L-Methionin (from Merk, Rio de Janeiro, Brazil)*.

e *DL-Lysine (from Merk, Rio de Janeiro, Brazil)*.

*Salt and vitamin composition are according to Horwitz et al. (1980)*.

### Tissue preparation

After the dietary experimental protocol, rats were sacrificed by decapitation, and blood was immediately collected in heparinized tubes and centrifuged at 2500 × g for 10 min at 20°C to yield the serum fraction, which was used for the subsequent biochemical analyses. Brains were quickly removed, and the hippocampus and cerebral cortex were dissected, weighed and either (i) cut into slices for substrate oxidation to CO_2_ or (ii) homogenized in a buffer of 0.32 M sucrose containing HEPES 1 mM, MgCl_2_ 1 mM, NaHCO_3_ 1 mM, phenyl-methyl-sulphonyl fluoride 0.1 mM, pH 7.4, in the presence of a complete set of protease inhibitors (Complete, Roche, Switzerland) for western blotting analysis (see description below).

### Blood samples and biochemical assays

The serum glucose, lactate (Labtest, MG, Brazil) and β-Hydroxybutyrate (BHB) (Cayman Chemical Company, MI, USA) levels were measured using commercial kits. Reactions were performed using the SpectraMax® Plus Microplate Spectrophotometer (Molecular Devices, CA, US).

### Substrate oxidation to ^14^CO_2_

To estimate lactate and BHB oxidation to ^14^CO_2_, 300-μm-thick hippocampal or cortical slices (weighing 40–60 mg), prepared with a McIlwain tissue chopper, were transferred into flasks and pre-incubated in a medium containing Krebs Ringer bicarbonate (KRB) buffer (pH 7.4) at 37°C for 30 min. Before incubation with substrates, the reaction medium was gassed with a 95% O_2_: 5% CO_2_ mixture for 30 s. Slices were incubated in 1 mL of KRB buffer containing either: (i) 10 mM sodium L-Lactate + 0.3 μCi L[U-^14^C] Lactate (56–186 mCi/mmol); or (ii) 10 mM DL-BHB sodium salt + 0.3 μCi [1-^14^C]-3-Hydroxybutyric acid sodium salt (50 mCi/mmol). Then, flasks containing the slices were sealed with rubber caps and parafilm, and incubated at 37 °C for 1 h in a Dubnoff metabolic shaker (60 cycles/min) as described previously (Ferreira et al., 2007). The incubation was stopped by adding 0.2 mL 50% tricarboxylic acid (TCA) through the rubber cap into the flask, while 0.1 mL of 2 N NaOH was injected into the central well. Thereafter, flasks were shaken for an additional 30 min at 37°C to trap CO_2_. Afterwards, the content of the central well was transferred to vials and assayed for radioactivity in a liquid scintillation counter. All results were calculated based on the initial radioactivity in the incubation medium and expressed in pmol/mg of tissue (Müller et al., 2013).

### Western blotting

Proteins (20 μg) were separated by SDS-PAGE on 10% (w/v) acrylamide and 0.275% (w/v) bisacrylamide gels and then electrotransferred onto nitrocellulose membranes according to (de Assis et al., 2015). Membranes were incubated for 12 h with the appropriate primary antibody (MCT1, 1:800; MCT2, 1:600; Pierre et al., 2000) and β-tubulin, 1:2000 (Santa Cruz Biotechnology, Heidelberg, Germany). Following the detection of chemiluminescent bands, densitometric analysis was performed using the Image-J® software.

### Statistical analyses

Data are expressed as mean ± S.E.M. All analyses were performed with the Statistical Package for the Social Sciences (SPSS 16.0—IBM, Chicago, IL, USA) software and Prism GraphPad Software (San Diego, CA, USA). Differences among groups were analyzed by one-way ANOVA and Tukey's *post-hoc* test or Kruskal-Wallis test followed by Dunn's Multiple Comparison test when necessary, with levels of significance below *P* < 0.05.

## Results

### Effects of the HAGE diet and/or DM on body parameters

We observed that the non-diabetic rats put on the HAGE diet had a significant (*P* < 0.05) increase in body weight and adipose tissue (Table 2). In the diabetic groups (D and D+HAGE) in contrast, we observed a reduction in body weight and adipose tissue for all rats, with reductions in the D group that were more pronounced (Table 2, *P* < 0.01).

**Table 2:** **Body weight and adipose tissue weight in control, HAGE fed, diabetic and diabetic HAGE fed rats**.

**Body parameters**	**C**	**HAGE**	**D**	**D+HAGE**
Initial body weight (g)	280±16.1	277±13.6	278±14.3	281±15.2
Final body weight (g)	315±20.1	332±21.2	229±33.9^*^	248±37.8^*^
Body weight gain (g)	35±7.2	55±11.8^*^	−49±12.6^**^	−33±9.5^**^
Adipose tissue (g)	3.4±0.3	8.4±2.9^**^	0.5±0.08^*^	4.6±1.4

Body weight and adipose tissue weight are expressed as grams (g). All results are presented as mean ± S.D. (n = 10 per group) and analyzed using one-way ANOVA and Tukey's post-hoc test. Adipose tissue is composed of epididymal + retroperitoneal adipose tissues. Asterisks indicate a significant difference compared to either the control group or the diabetic group for the HAGE group and D+HAGE group, respectively (

* P < 0.05,

** P < 0.01, ***P < 0.001).

### Effects of the HAGE diet and/or DM on blood biochemical profile

The plasmatic glucose levels were significantly elevated in both diabetic groups (~3.5-fold, *P* < 0.001 vs. respective control groups, Table 3; The HAGE diet had no effect *per se*). The plasmatic lactate levels were not affected by diet or DM (Table 3). In contrast, the plasmatic BHB levels were not affected by DM induction alone compared to control animals, but they were significantly higher in rats fed with the HAGE diet (~3.0-fold vs. control, *P* < 0.05, Table 3) with a stronger effect of the HAGE diet in diabetic rats compared to control animals (~13-fold vs. DM alone, *P* < 0.01; *P* < 0.01 vs. HAGE, Table 3). The rats submitted to the HAGE diet or diabetes induction showed higher levels of triglycerides (Table 3, *P* < 0.05) and for the rats submitted to both protocols this increase is more marked (Table 3, *P* < 0.01). We found no significant difference in plasma cholesterol, HDL and free fatty acids of the groups tested.

**Table 3:** **Blood biochemical profile of control, HAGE fed, diabetic and diabetic HAGE fed rats**.

**Blood biochemical profile**	**C**	**HAGE**	**D**	**D+HAGE**
Glucose (mmol/L)	5.27±0.47	5.43±0.57	22.78±4.86^***^	20.48±2.13^***^
Lactate (mmol/L)	3.84±0.93	4.65±0.69	3.79±0.65	4.44±0.70
β-hydroxybutyrate (mM)	0.42±0.20	1.46±0.42^*^	0.46±0.16	4.32±0.45^**^^,^ ^#^
Triglycerides (mg/dL)	70.9±20.1	144.1±74.8^*^	140.1±56.2^*^	258.6±69.1^**^^,^ ^#^
Cholesterol (mg/dL)	64.8±11.2	76.1±25.9	76.1±16.4	71.5±10.4
HDL (mg/dL)	23.5±5.3	25.5±5.7	26.0±4.8	25.6±2.5
Free Fatty Acids (μM)	566.1±60.5	523.6±126.5	583.0±45.1	616.4±227.6

All results are presented as mean ± S.D. (n = 10 per group) and analyzed using one-way ANOVA and Tukey's post-hoc test. Asterisks indicate a significant difference compared to either the control group or the diabetic group for the HAGE group and D+HAGE group, respectively (

* P < 0.05,

** P < 0.01,

*** P < 0.001). Plus sign indicates a significant difference between the HAGE and D+HAGE groups (

# *P < 0.05)*.

### Effects of HAGE diet and/or DM on lactate and BHB oxidation and on MCT1 and MCT2 protein expression in the hippocampus and cerebral cortex

**Figure 1:**
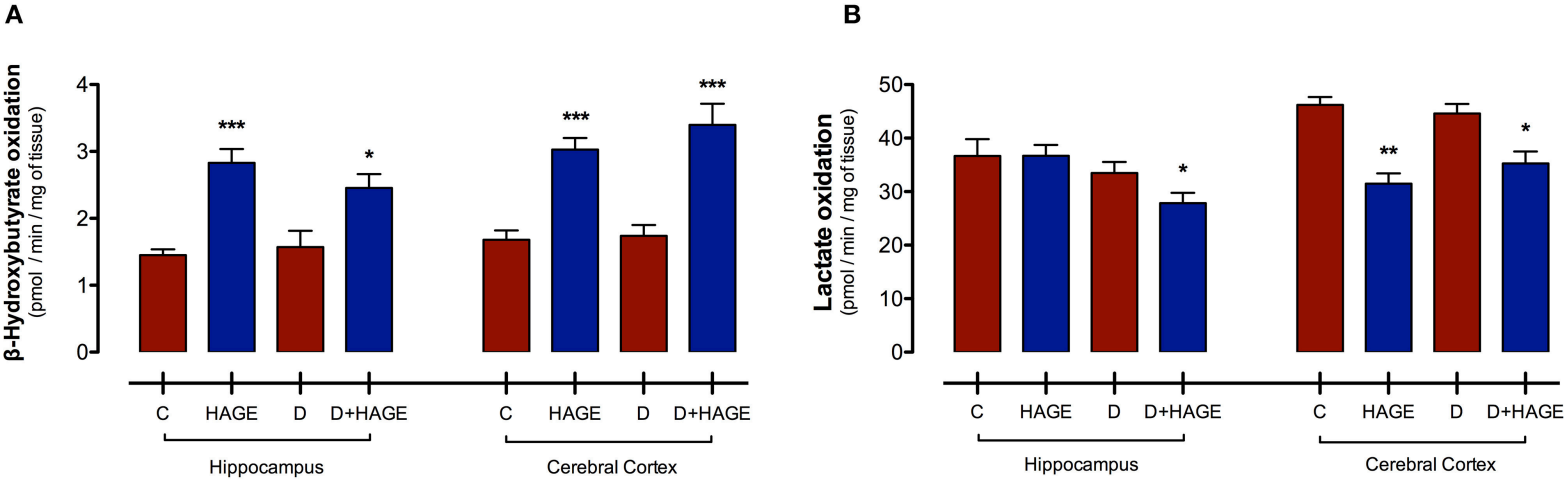
**Effects of the HAGE diet and/or DM on lactate and BHB oxidation in the hippocampus and cerebral cortex. (A)** Lactate oxidation to CO_2_ in rat hippocampal and cortical slices; and **(B)** BHB oxidation to CO_2_ in rat hippocampal and cortical slices. Lactate and BHB oxidation are expressed as pmol of substrate oxidized to ^14^CO_2_/min/mg of tissue. The results are presented as mean ± S.E.M. (*n* = 10 per group for oxidation experiments) and analyzed using one-way ANOVA and Tukey's *post-hoc* test. Asterisks indicate a significant difference compared to either the control group or the diabetic group for the HAGE group and D+HAGE group, respectively (*P < 0.05, **P < 0.01, ***P < 0.001).

Interestingly, monocarboxylic acid oxidation to CO_2_ was differently affected by the HAGE diet and/or DM, depending on the brain structure and the substrate examined. The oxidation rate of BHB was increased by the HAGE diet in both structures, (*P* < 0.001 vs. control and D groups for both structures; *P* < 0.05, D+HAGE vs. control and D groups for hippocampus; *P* < 0.001, HAGE and D+HAGE vs. control and D groups for cerebral cortex, Figure 1A), but DM alone had no effect. Lactate oxidation was decreased by the association of DM induction and the HAGE diet in both brain structures as well as by the HAGE diet alone in the cerebral cortex (*P* < 0.05, D+HAGE vs. all groups for hippocampus; *P* < 0.01, HAGE vs. control and D groups and *P* < 0.05, D+HAGE vs. control and D groups for cerebral cortex) (Figure 1B).

**Figure 2:**
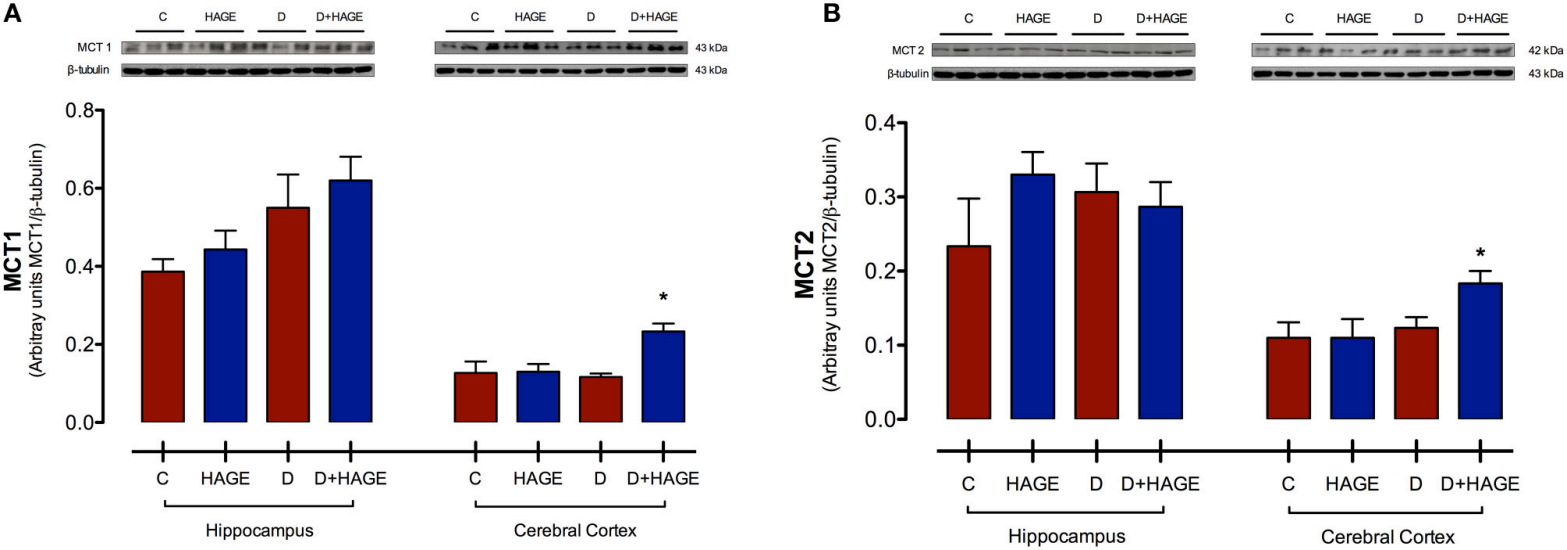
**Effects of the HAGE diet and/or DM on MCTs immunocontent in the hippocampus and cerebral cortex. (A)** Representative and quantitative Western blot analysis of MCT1 protein expression in rat hippocampus and cerebral cortex; **(B)** Representative and quantitative Western blot analysis of MCT2 protein expression in rat hippocampus and cerebral cortex. β–tubulin was used as reference. The results are presented as mean ± S.E.M. (*n* = 3 per group) and analyzed using Kruskal-Wallis test followed by Dunn's Multiple Comparison test. Asterisks indicate a significant difference compared to either the control group or the diabetic group for the HAGE group and D+HAGE group, respectively (*P < 0.05).

Protein expression levels of MCT1 and MCT2 were significantly upregulated only in the cerebral cortex with both DM induction and the HAGE diet, but not by either condition alone (MCT1 in the cerebral cortex, *P* < 0.05, D+HAGE vs. all groups, Figure 2A; MCT2 in the cerebral cortex, *P* < 0.05, D+HAGE vs. all groups, Figure 2B). Similarly, we can see an increase in protein expression levels of MCT1 and MCT2 in the hippocampus of diabetic rats fed the HAGE diet, however there was no statistical difference between the groups.

## Discussion

The main results in this study show that rats fed with a high fat diet enriched with AGEs presented (i) a simultaneous increase in serum BHB levels and its hippocampal and cortical oxidation rate; (ii) a decrease in lactate oxidation for both structures in the diabetic, HAGE diet-fed group; and (iii) an increase in MCT1 and MCT2 expression in the cerebral cortex, but only in the diabetic, HAGE diet-fed group. This is the first investigation suggesting a putative impact of a high fat, AGE-enriched diet on brain energy metabolism that occurs by modifying the availability and utilization of specific substrates and the expression levels of some brain MCT isoforms.

Our data show that diabetes induced by the destruction of pancreatic β-cells caused an expected increase in blood glucose (Lenzen, 2008) but produced no change in the levels of blood lactate or blood ketone bodies. It may seem surprising that a ketotic state did not develop following the destruction of pancreatic β cells, as previously reported (Miethke et al., 1986; Rösen et al., 1986; Kante et al., 1990). However, it has also been reported that the induction of DM type I with alloxan in rats may only lead to the partial destruction of pancreatic β cells, depending on the dose and administration regimen, and may thus cause the induction of type II diabetes mellitus (Federiuk et al., 2004). In such cases, it has been reported that rats do not spontaneously develop a ketotic state. In parallel, exposure to the HAGE diet had no impact on blood glucose or lactate levels, while it enhanced blood ketone body levels as expected (Crane and Morgan, 1983). The combination of diabetes and exposure to the HAGE diet had no effect on the blood lactate levels and no further effect on the blood glucose levels. However, it produced a stronger elevation in the blood ketone body levels. This observation would be consistent with insufficient levels of insulin. In this situation, lipogenesis is reduced and ketogenesis by the liver is favored (Lombardo et al., 1978; Roman-Lopez and Allred, 1987). The progressive formation of ketone bodies from fatty acids supplied by the HAGE diet can then occur.

In contrast to its peripheral effects, little information about the impact of diabetes and high fat diets on brain metabolism is available to date. Our results suggest that exposure to a HAGE diet causes profound modifications in monocarboxylate utilization in both hippocampal and cortical tissue. Both lactate and ketone body utilization depend on their uptake into brain cells via monocarboxylate transporters (Carneiro and Pellerin, 2015). Previously, it has been shown that long-term exposure to a regular ketogenic diet leads to an enhancement of the expression of monocarboxylate transporters in the central nervous system (Leino et al., 2001; Pierre et al., 2007; Puchowicz et al., 2007). In our case, no enhancement in either MCT1 or MCT2 expression could be observed in either the hippocampus or cortex. Possible explanations could be either the duration of diet exposure, as in the study of Pierre et al., it took 12 weeks of exposure to a high fat diet to obtain significant increases in MCT expression (Pierre et al., 2007), or the composition of the diet itself, as the presence of AGEs might also play a role. Nevertheless, an interesting observation is the fact that the combination of diabetes and exposure to the HAGE diet led to a significant enhancement in the expression of MCT1 and MCT2 in the cortex. This is surprising because it has previously been shown that MCT2 expression in cultured cortical neurons is enhanced by insulin (Chenal et al., 2008). The absence of peripheral insulin might have favored the emergence of other factors, which could have been triggered by the exposure to the HAGE diet. Apart from insulin, several other neuroactive substances have been identified that could modulate monocarboxylate transporter expression, including BDNF (Robinet and Pellerin, 2010, 2011), IGF1 (Chenal et al., 2008), noradrenaline (Chenal and Pellerin, 2007), and nitric oxide (Marcillac et al., 2011).

Although the increase in monocarboxylate transporter expression in our experiment might have facilitated the consumption of ketone bodies in diabetic animals, it is unlikely to be the limiting factor, as the exposure of normal rats to the HAGE diet led to similar changes in ketone body oxidation. We must postulate that the HAGE diet could have altered the expression and/or activity of key enzymes for ketone body metabolism, such as d-β-hydroxybutyrate dehydrogenase. Indeed, it has been previously reported in situations such as starvation and hyperlipidemic diet in rats, concomitant with an increase in availability of blood ketone bodies, that there is an increase in the cerebral expression and activity of D-β-hydroxybutyrate dehydrogenase (Kante et al., 1990; Leino et al., 2001). Similarly, the reduction in lactate oxidation observed in diabetic animals treated with the HAGE diet could be due to alterations in lactate dehydrogenase expression and/or activity. In fact, it has been reported previously that in rats treated with alloxan, a reduction in cerebral lactate dehydrogenase activity could be observed (Ahmed and Zahra, 2011). This switch in the use of different oxidative substrates (from lactate to ketone bodies) by brain cells might constitute an attempt to overcome the deleterious effects of AGEs on some unknown component of their energetic metabolism. It is likely that the impact of diabetes and AGEs on the metabolism of neurons and astrocytes is different. However, more investigations are needed to determine how each cell type is affected, and what are the global consequences for brain metabolism. Interestingly, it was recently reported that the replacement of glucose by ketone bodies as energy source for neurons allows to maintain synaptic vesicle recycling but it slows down both exocytosis and endocytosis (Hrynevich et al., 2016). Indeed, a preferential use of ketone bodies in these circumstances would fit well with the reported protective effects of ketogenic diets in neurodegenerative diseases and epilepsy (Hartman, 2012).

In conclusion, the present study provides preliminary evidence that a high fat diet enriched with AGEs (control or diabetes groups) given for 4 weeks led to an increase in the serum levels of ketone bodies and simultaneously increased their use as energy sources in the hippocampus and the cerebral cortex. Interestingly, only the combination of a HAGE diet and DM induction increased the cerebral expression of the two MCT isoforms only in cerebral cortex. In parallel, the combination of DM and the HAGE diet also caused a decrease in lactate oxidation. These results suggest that our dietary manipulation together with DM type I induction favored BHB instead of lactate as an energy source for brain cells. Further studies should be performed to better understand the cellular mechanisms involved in the brain alterations induced by the combination of a high fat diet enriched with AGEs and DM.

## Author contributions

AD was responsible for the design, acquisition, analysis, interpretation, drafting, and approval of the final version of the manuscript. JD, AR, AL, YN, CR, and MD were responsible for acquisition, analysis, interpretation, and approval of the final version of the manuscript. JM and DS were responsible for interpretation, drafting, critical revision, and approval of the final version of the manuscript. LP was responsible for the design, interpretation, drafting, critical revision, and approval of the final version of the manuscript.

### Conflict of interest statement

The authors declare that the research was conducted in the absence of any commercial or financial relationships that could be construed as a potential conflict of interest.
